# Experimentally induced and real-world anxiety have no demonstrable effect on goal-directed behaviour

**DOI:** 10.1017/S0033291720000203

**Published:** 2021-07

**Authors:** C. M. Gillan, M. M. Vaghi, F. H. Hezemans, S. van Ghesel Grothe, J. Dafflon, A. B. Brühl, G. Savulich, T. W. Robbins

**Affiliations:** 1 Trinity College Dublin, Dublin, Ireland; 2 New York University, New York, USA; 3 University of Cambridge, Cambridge, UK; 4 University of Amsterdam, Amsterdam, Netherlands; 5 Kings College London, London, UK; 6 University Hospital of Psychiatry Zurich, Zurich, Switzerland

**Keywords:** Anxiety, Goal-directed control, Obsessive-compulsive disorder

## Abstract

**Background:**

Goal-directed control guides optimal decision-making and it is an important cognitive faculty that protects against developing habits. Previous studies have found some evidence of goal-directed deficits when healthy individuals are stressed, and in psychiatric conditions characterised by compulsive behaviours and anxiety. Here, we tested if goal-directed control is affected by state anxiety, which might explain the former results.

**Methods:**

We carried out a causal test of this hypothesis in two experiments (between-subject *N* = 88; within-subject *N* = 50) that used the inhalation of hypercapnic gas (7.5% CO_2_) to induce an acute state of anxiety in healthy volunteers. In a third experiment (N = 1413), we used a correlational design to test if real-life anxiety-provoking events (panic attacks, stressful events) are associated with impaired goal-directed control.

**Results:**

In the former two causal experiments, we induced a profoundly anxious state, both physiologically and psychologically, but this did not affect goal-directed performance. In the third, correlational, study, we found no evidence for an association between goal-directed control, panic attacks or stressful life eventsover and above variance accounted for by trait differences in compulsivity.

**Conclusions:**

In sum, three complementary experiments found no evidence that anxiety impairs goal-directed control in human subjects.

## Background

Two well-established systems contribute to everyday decision making and behaviour, the goal-directed and the habitual system (Dickinson, 1985). Goal-directed behaviour is characterised by actions that are appropriate to the current desire for a given outcome and informed by the knowledge of the causal relationship between an action and the associated outcome (Dickinson & Balleine, 1994). More recently goal-directed control has been formalised as model-based planning, within a reinforcement learning framework (Daw, Gershman, Seymour, Dayan, & Dolan, 2011).

Though no previous study has examined whether experimentally induced state anxiety impairs goal-directed planning, a related literature on stress-induction offers a basis for this suggestion. Specifically, acute stress has been shown to induce deficits in goal-directed planning (Park, Lee, & Chey, 2017; Schwabe & Wolf, 2009, 2010), albeit inconsistently (null results: Heller, Ezie, Otto, & Timpano, 2018; Otto, Raio, Chiang, Phelps, & Daw, 2013; Radenbach et al. 2015) in healthy individuals. Acute anxiety and stress manipulations produce similar cardiovascular changes, and induce negative affect, but anxiety induction differs from stress in terms of the specific psychological experience (e.g. increased vigilance, panic, fear) and other aspects of the physiological response (Bailey, Argyropoulos, Kendrick, & Nutt, 2005; Shin & Liberzon, 2010).

Physiological and psychological stress has been likened to anxiety, and it is generally thought to impair several forms of deliberative and reflective processes, in favour of more automatic and reflexive ones (Shields, Sazma, & Yonelinas, 2016). From a neurobiological perspective, there is evidence that this mechanism is regulated by catecholamines, which act on prefrontal functioning under stress (Arnsten 1998). It has been suggested that reliance on faster, habitual mechanisms might be an evolutionary advantage in stressful situations (Arnsten 1998). Similarly, in the case of anxiety, the attentional control theory (Eysenck et al., 2007) suggests that anxiety impairs cognitive performance of top-down, executive tasks by giving greater influence to the bottom-up attentional system.

In addition, anxiety is a prominent feature of pathological manifestations characterised by an impoverished goal-directed system. For example, a fragile goal-directed system is hypothesised to lead one to get stuck in habits (Gillan, Otto, Phelps, & Daw, 2015) and typifies not only Obsessive-Compulsive Disorder (OCD) (Gillan et al., 2011; Gillan & Robbins, 2014; Vaghi et al., 2018) but also several other psychiatric conditions on the compulsivity spectrum such as eating disorder, drug abuse and alcohol addiction (Sjoerds et al., 2013; Voon et al., 2014). Accordingly, it has been suggested that goal-directed deficits constitute a trans-diagnostic trait (Gillan, Kosinski, Whelan, Phelps, & Daw, 2016; Robbins, Gillan, Smith, de Wit, & Ersche, 2012). One potential issue with this model is its specificity. Compulsivity is highly comorbid with anxiety (Nestadt et al., 2009), which is unsurprising, as OCD has only recently moved out of the Diagnostic and Statistical Manual category of anxiety disorders into its own classification (Stein et al., 2010). Accordingly, this raises the possibility that elevated anxiety levels in OCD might account for failures in goal-directed planning and consequent overreliance on habits.

In support of this idea, social anxiety patients appear to show similar deficits in goal-directed planning to OCD patients, despite the fact that they do not have a compulsive phenotype (Alvares, Balleine, & Guastella, 2014). Cross-sectional, correlational work has started to address this issue, finding that when a range of psychopathology measures are taken (and controlled for) within the same individuals, there is no meaningful contribution of trait anxiety to goal-directed deficits, while the association with compulsivity is robust (Gillan et al., 2016; Robbins et al., 2012). However, these studies are limited not just by their correlational nature, but because they assess trait anxiety, which does not speak to acute states of anxiety that are experienced by patients more transiently, often in response to their own obsessive and compulsive symptoms (Mataix-Cols et al., 2003).

Here, we aimed to characterise the relationship between increased anxiety and the functioning of the goal-directed system. We used a combination of causal and correlational approaches to investigate this in three experiments spanning laboratory and real-life settings.

Firstly, we used hypercapnic gas (i.e. with increased CO_2_ level) to experimentally induce state anxiety and test its impact on goal-directed control, operationalised as sensitivity to contingency degradation (Vaghi et al., 2018). Hypercapnic gas is a well-validated method for experimentally inducing a transitory state of acute anxiety in healthy volunteers (Woods, Charney, Goodman, & Heninger, 1988). At very high doses (35% CO_2_) it generates symptoms similar to those of panic disorder, with increased blood pressure and bradycardia (Argyropoulos et al., 2002; Griez, Zandbergen, Pols, & de Loof, 1990; Perna, Barbini, Cocchi, Bertani, & Gasperini, 1995), especially in subjects with panic disorder or susceptibility to it (Perna, Bertani, Caldirola, & Bellodi, 1996; Perna et al., 1994). We used lower doses (7.5% CO_2_) which are reported to be sufficient to induce physiological and psychological symptoms of anxiety and sustained arousal associated with an anxiety state (Bailey et al., 2005). Subjects had profound physiological and subjective psychological responses to the anxiety induction procedure including changes in the heart rate, blood pressure and self-reported anxiety, but it failed to induce deficits in goal-directed control over behaviour.

To attempt to rule out the possibility that this null effect was an issue of the sensitivity of our study design, we repeated this experiment using a within-subjects design and a more commonly used, and potentially more sensitive, measure of goal-directed control – a ‘model-based planning’ measure derived from the two-step reinforcement learning task described above (Daw et al., 2011). Again, the CO_2_ manipulation resulted in substantial physiological and psychological effects consistent with the induction of an acute state of anxiety, but this had no demonstrable detrimental effect on goal-directed behaviour.

In a third and final experiment, we tested this hypothesis in a naturalistic, real-world setting using a large-scale correlational design (*N* = 1413) (Gillan et al., 2016). We investigated if goal-directed (model-based) control is impaired in individuals who suffered recent ‘real life’ acute anxiety, specifically known to be associated with the experience of a recent panic attack (Aronson & Logue, 1988) and/or major life-stressors (Vyas, Pillai, & Chattarji, 2004). We found that the frequency of panic attacks in the past week and higher levels of stress in the past year were both modestly associated with deficits in goal-directed planning. Crucially, neither survived controlling for a correlated psychiatric trait, compulsive behaviour and intrusive thought, which we previously showed have a strong association with goal-directed planning using these same data (Gillan et al., 2016).

## Methods

### Experiment 1

#### Subjects

A total of 88 participants was recruited through university mailing lists, departmental research panels and posted flyers within the University of Cambridge and the wider community. Participants were randomly assigned to either the CO_2_-induced anxiety group (*n* = 43, 20 females; mean age = 27.55, s.d. = 11.04) or the normal air ‘placebo’ group (*n* = 45, 24 females; mean age = 27.40, s.d. = 10.03) (online Supplementary Material for further details on recruitment and inclusion and exclusion criteria).

#### Anxiety manipulation

The anxiety induction consisted of the inhalation of air enriched with 7.5% CO_2_ (7.5% CO_2_, 20% O_2_, 71.5% N_2_, pre-mixed, BOC Special Gases, Guildford, UK). As the experimenter had to manually switch a lever to activate the delivery of one of the two air preparations, CO_2_ was administered in a single-blind manner while measuring goal-directed/habit behaviour via controlled tasks, and was designed to induce a physiological state of acute anxiety in a reliable and controlled manner (Bailey et al., 2005). Participants inhaled the assigned air preparation as long as they were doing the task. To measure the effectiveness of this procedure at inducing acute anxiety, we recorded physiological measurements comprising heart rate, diastolic and systolic blood pressure and psychological measurements comprising the 17-item Acute Panic Inventory (Liebowitz et al., 1984), 10-item Positive and Negative Affective Scale (Watson, Clark, & Tellegen, 1988) and three Visual Analogue Scales assessing anxiety, fear and happiness. Physiological measures were collected 10 min before, during and 15 min after the experimental manipulation. Psychological measures of subjective feeling due to the experimental manipulation were concomitantly collected, the only difference being that they were not interrogated during the performance of the task but immediately after and retrospectively on how they were feeling.

#### Contingency degradation paradigm

**Fig. 1.**
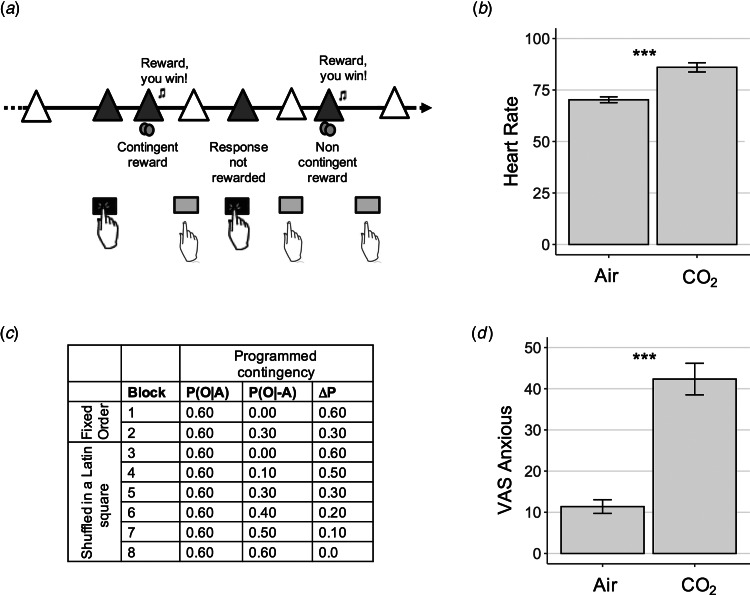
Experiment 1 Study Design – Contingency Degradation Task. (*a*) Contingency degradation task design. In each block, subjects were presented with a white triangle, signalling that they had the opportunity to press or to not press the space bar, in a free-operant, self-paced procedure (Vaghi et al., 2018). The triangle turned yellow (here pictured in grey) when a response was recorded. Rewards (a 25 pence image) were delivered according to a probability of outcome given action, P(O|A), on trials when a response was made, and a probability of outcome given no action, P(O|−A), when a response was not made. (*b*) Physiological response to anxiety induction. Subjects' heart rate was elevated significantly during the gas condition, *p* < 0.001. Error bars represent s.e.. (*c*) Programmed contingencies. Each participant completed eight blocks where contingency was systematically varied through changes to P(O|−A). The first two blocks were considered training blocks and appeared in a fixed order as denoted in the table. The six remaining test blocks were presented in a counterbalanced order across subjects. (*d*) Psychological response to anxiety induction. Anxiety scores measures using a visual analogue scale (VAS) were also significantly elevated during the inhalation of gas compared with air, *p* < 0.001. Error bars represent s.e. ***, *p* < 0.001.

In this between-subjects design, subjects in each group performed a contingency degradation task described previously and further detailed in the online Supplementary Material (Vaghi et al., 2018) (Fig. 1*a*). In short, the task was a free operant, self-paced procedure which tests subjects' ability to detect action-outcome instrumental contingencies (Vaghi et al., 2018), one of the earliest operationalisations of goal-directed learning from the animal literature (Dickinson, Nicholas, & Adams, 1983).

#### Experienced contingency

As expected, for normal and CO_2_-enriched air condition, experienced contingencies (based on experienced event frequencies, see online Supplementary Material, Table S1) matched the a priori programmed ones (CO_2_: *r* = 1.00, *p* < 0.001; air: *r* = 1.00, *p* < 0.001). Therefore, programmed contingencies were used for subsequent analysis. Our findings were not confounded by between-group differences in experienced contingencies, as no main effect of group (*F*_(1, 63)_ = 0.80, *p* = 0.37, *η*^2^_G_ = 0.003) nor interaction between group and block (*F*_(2.57, 161.61)_ = 0.17, *p* = 0.89, *η*^2^_G_ = 0.002) was found.

#### Data analysis

We first performed analyses of variance (ANOVA) to determine whether there was a between-group difference in sensitivity to instrumental contingency as measured by the response rate and causality judgment. The response rate was computed by dividing the number of bins for which a response was made by the total number of bins within each block. For each dependent variable, programmed contingency was used as a within-subject factor, and group was used as a between-subject factor. Analyses were conducted separately for the initial learning blocks and the test blocks. For the test blocks, we also investigated the relationship between the response rate and contingency judgments, using a linear mixed-effects model. Specifically, we used contingency judgement and group as fixed effects, and we allowed the intercept and slope to vary between participants as random effects. We obtained *p* values for the fixed effects using the Kenward–Roger method. Bayes factor analysis was used in case of failure to reject the null hypothesis, to examine the relative evidence for the null with default JZS priors for ANOVA (Rouder, Speckman, Sun, Morey, and Iverson, 2009) and (Rouder, Morey, Speckman, & Province, 2012). Previous research (Schwabe, Tegenthoff, Höffken, & Wolf, 2010) found a between-subjects effect size of stress on habitual performance for which default JSZ priors are suitable as specified in Rouder et al., 2009 and Rouder et al., 2012. Analyses were performed in R version 3.4.3 (R Foundation for Statistical Computing, Vienna, Austria; http://www.r-project.org/) using the ‘afex’ package for ANOVA and linear mixed models, the ‘Bayes Factor’ and ‘brms’ package for Bayes factor analysis and the ‘tidyverse’ packages for data organisation and visualisation.

### Experiment 2

#### Subjects

A total of 61 healthy volunteers was recruited from the local community in the same manner as described in Experiment 1. Screening and exclusion criteria were identical to Experiment 1. Further exclusion criteria were applied contingent on the experimental task employed here (online Supplementary Material). The final sample size for analysis was 50 (26 female) with ages ranging from 18–62.

#### Reinforcement learning task

Participants completed a reinforcement-learning task that quantifies individual differences in goal-directed (‘model-based’) learning, which is operationalised as a parameter estimate from a logistic regression analysis predicting choices in the task (Daw, Niv, & Dayan, 2005). The task (Fig. 3*a*) has been extensively used and described elsewhere (37) and further detailed in the online Supplementary Material.

#### Anxiety induction

The anxiety induction procedure as well as collection of physiological and psychological measures was identical to Experiment 1, except for the within-subjects design. Participants attended a single test session during which they completed two versions of the Reinforcement Learning Task during 20 min inhalation of air enriched with 7.5% CO_2_ and normal air. Gas was administered in a single-blind manner and the order of CO_2_ *v.* normal air was counterbalanced.

#### Data analysis

Data were analysed using mixed-effects logistic regression in the *lme4* package in R 3.5.1 (http://cran.us.r-project.org). In line with previous studies (Daw et al., 2011), we tested the extent to which subjects tend to repeat actions performed on the previous trial or explore a new one (‘Stay’: coded switch = 0; stay = 1), and whether these choices were influenced by whether or not their previous action was rewarded (‘Reward’: coded as rewarded = 1; unrewarded = −1), was followed by a rare or common transition (‘Transition’: coded as common = 1, rare = −1) and their interaction (‘Reward × Transition’). The intercept reflects tendencies to repeat the same action from one trial to the next, the main effect of reward reflects the contribution of model-free learning to subjects' choices, while an interaction between reward and transition is the hallmark of model-based (goal-directed) behaviour. We included anxiety induction as a within-subjects factor (coded CO_2_ = 1, Air = −1). We used Bound Optimization by Quadratic Approximation with 1e5 functional evaluations. The model was specified as follows: Stay ~ Reward × Transition × CO_2_ + (Reward × Transition × CO_2_ + 1|Subject). Bayes factor analysis was used in case of failure to reject the null hypothesis using the anovaBF function in the BayesFactor package in R, with default JZS priors for ANOVA from (Rouder et al., 2012). To avoid the issues with nested interactions from the logistic model, we extracted estimates for model-based planning separately for each subject in each condition and used these to compare an ANOVA model with a within-subjects effect of gas to an intercept-only model.

#### Computational modelling

A more elaborated form of this analysis is presented in the online supplement. In brief, this method allows for analysis of a greater number of potential behavioural confounds, including separating the distinct role of the learning rate and choice randomness from that of model-based, model-free and choice repetition estimates from the simpler analysis. These results largely recapitulate the main findings of the paper, with slight differences flagged as appropriate.

### Experiment 3

#### Participants

Data were collected online using Amazon's Mechanical Turk. Details of the experimental procedure can be found elsewhere (Gillan et al., 2016), but in brief, data were analysed from 1413 individuals (823 female) with ages ranging from 18 to 76 (M = 33, s.d. = 11), who were based in the USA, had a history of good performance on the platform (i.e. were paid in full on at least 95% of their previous tasks), passed a comprehension test, and did not fall victim to a ‘catch’ question (online Supplementary Material).

#### Reinforcement learning task

The task employed in this study was the same as that described in Experiment 2. The only difference was that subjects completed it remotely, and therefore a more rigorous quality control procedure was implemented (detailed in Supplement).

#### Panic attacks and life stress

The occurrence of recent panic attacks was assessed using item 1 on the self-report version of the Panic Disorder Severity Scale (PDSS; Shear et al., 1997). Life stress was assessed using the Social Readjustment Scale (Holmes & Rahe, 1967), which presents an inventory of common stressful life events to participants and asks them to select those that applied to them in the previous 12 months. The present sample had a mean score of 159 (s.d. = 120). Scores lower than 150 are considered evidence of ‘no significant stress’ (*N* = 775), while scores in excess of 300 are considered signs of major stress (*N* = 179 in this sample) (Fig. 5*b*) (see also online Supplementary Material). Control variables were also included as detailed in the online Supplementary Material.

#### Data analysis

We performed the same analysis as in Experiment 2, but here we additionally controlled for variables that have been previously linked to model-based planning, namely: IQ, age, gender and a trans-diagnostic psychiatric trait ‘Compulsive Behaviour and Intrusive Thought’. This covariate was derived from previous published work (Gillan et al., 2016; Rouault, Seow, Gillan, & Fleming, 2018) that applied factor analysis to a series of questionnaires linked to self-reported measures of psychopathology. Factors were labelled based on items that loaded most strongly on each of the identified factors. Accordingly, items from questionnaires related to ‘compulsive’ disorders such as OCD and addiction most strongly loaded on the factor named ‘Compulsive Behaviour and Intrusive thought’. Scores of each subject on this factor were used as a covariate in the present analysis to rule out the possibility that any association between stress, panic attacks and goal-directed control could be better explained by compulsivity. Bayes factor analysis was conducted on a linear model where residuals for model-based planning was the dependent measure and life stress or panic symptoms were the experimental models compared to an intercept-only model. As in experiment 2, we complemented our regression analysis with a computational model, details of which are available in the online supplement.

## Results

### Anxiety induction and contingency degradation (Experiment 1)

Here we tested if experimentally induced anxiety would affect subjects' ability to detect action-outcome instrumental contingencies. In a between-subjects design, one group was assigned to inhale hypercapnic gas (7.5% CO_2_) during the performance on the contingency degradation task, while the other inhaled normal air. Psychological and physiological measures confirmed that anxiety induction was successful and of a magnitude similar to that observed in prior studies (Cooper et al., 2013; Garner, Attwood, Baldwin, & Munafò, 2012; Garner, Attwood, Baldwin, James, & Munafò, 2011): participants in the CO_2_ condition experienced greater self-reported anxiety (*F*_(1.97, 159.61)_ = 35.57, *p* < 0.001) and had a higher heart rate (*F*_(1.96, 152.92)_ = 36.64, *p* < 0.001) than those assigned to the air condition (Fig. 1*b* and *d*; online Supplementary materials).

**Fig. 2.**
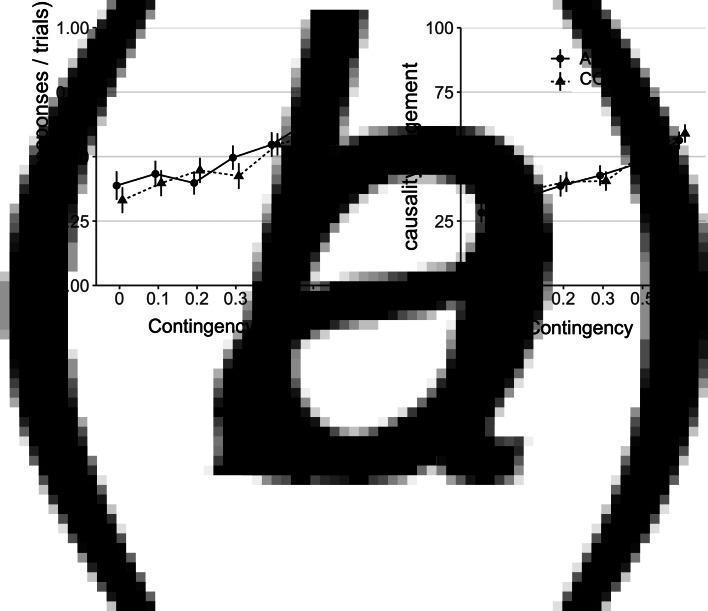
Results from Experiment 1. (*a*) There was no effect of CO_2_-induced anxiety on subjects' sensitivity to instrumental contingency as measured by choice responses, *F*_(3.73, 320.59)_ = 1.74, *p* = 0.15. Error bars represent s.e. (*b*). There was similarly no effect of group on the extent to which causality judgements scaled with instrumental contingency, *F*_(2.99, 256.89)_ = 0.33, *p* = 0.81. Error bars represent s.e.

Participants learnt the contingencies in the training phase (*F*_(1, 86)_ = 26.48, *p* < 0.001, *η*^2^_G_ = 0.03). Experimentally induced anxiety did not affect subjects' behavioural sensitivity to instrumental contingency. Participants overall adjusted their response rate in line with the underlying contingency, as evidenced by a main effect of contingency on the response rate in the test blocks (*F*_(3.73, 320.59)_ = 29.95, *p* < 0.001, *η*^2^_G_ = 0.07). In the test blocks, there was no between-group difference (*F*_(1, 86)_ = 0.22, *p* = 0.64, *η*^2^_G_ = 0.002) and no group by contingency interaction (*F*_(3.73, 320.59)_ = 1.74, *p* = 0.15, *η*^2^_G_ = 0.004) (Fig. 2*a*). Bayes factor analysis further confirmed these findings. Specifically, the null model was strongly preferred over the alternative model with the main effect of anxiety and interaction effect of anxiety by contingency (BF_01_ = 16.81 and online Supplementary Fig. S1*a*).

The same was true of participants' *subjective* assessments of instrumental contingency (i.e. their explicit model of the environment). Subjects accurately tracked the underlying contingency of the task (training blocks, *F*_(1, 86)_ = 30.46, *p* < 0.001, *η*^2^_G_ = 0.12; test blocks, *F*_(2.99, 256.89)_ = 26.22, *p* < 0.001, *η*^2^_G_ = 0.13) and the experimental manipulation did not affect this. There was no between-group difference (*F*_(1, 86)_ = 0.16, *p* = 0.69, *η*^2^_G_ = 0.001) and no group by contingency interaction (*F*_(2.99, 256.89)_ = 0.33, *p* = 0.81, *η*^2^_G_ = 0.002) (Fig. 2*b*) on causality judgements. Bayes factor analysis further confirmed these findings. Specifically, the null model was strongly preferred over the alternative model with a main effect of anxiety and interaction effect of anxiety by contingency (BF_01_ = 386.15 and online Supplementary Fig. S1*b*). Mirroring the findings on choice responses, experimentally induced anxiety did not affect subjective judgments of instrumental contingency – adding weight to the suggestion that state anxiety may not have an appreciable effect on goal-directed control over action.

### Individual differences

Prior work showed that individual differences might be important in revealing the effect of stress on goal-directed behaviour (Heller et al., 2018; Otto et al., 2013; Radenbach et al., 2015; Schwabe & Wolf, 2010). Therefore, we tested if the detrimental effect of CO_2_ on goal-directed behaviour might depend on individual differences in sensitivity to the CO_2_ manipulation, assessed in terms of change in both self-reported and physiological measures of anxiety. For the former, we ran the model explained above with programmed contingency as a within-subject factor, introducing a change in self-report anxiety as a between-subject covariate. The change in self-report anxiety was computed as the difference between VAS-anxious before inhaling the gas and after inhaling the gas. As above, there was a significant effect of programmed contingency on the response rate (*F*_(3.73, 309.85)_ = 25.42, *p* < 0.001), but there was no main effect of subjectively reported change in self-report anxiety (*F*_(1, 83)_ = 0.28, *p* = 0.60) nor an interaction effect with programmed contingency (*F*_(3.73, 309.85)_ = 0.20, *p* = 0.42). Similar findings were obtained on subjective causality ratings. Accordingly, programmed contingency significantly predicted causality ratings (*F*_(3.18, 264.24)_ = 33.10, *p* < 0.001), but there was not a main effect (*F*_(1, 83)_ = 0.00, *p* = 0.96) nor a significant interaction with subjectively reported change in self-report anxiety (*F*_(3.18, 264.24)_ = 0.20, *p* = 0.90). Therefore, individual differences in anxiety, as self-reported by subjects upon CO_2_ challenge, did not affect goal-directed planning.

We conducted the same analyses by using physiological changes in the heart rate as a putatively more objective measure of the change in anxiety arising from our manipulation. The physiological index for change in the heart rate was computed as above, i.e. the difference between the heart rate before inhaling the gas and after inhaling the gas. Changes in the heart rate did not have a main effect on the response rate (*F*_(1, 80)_ = 0.1, *p* = 0.75), but there was a trend for an interaction between changes in the heart rate and programmed contingency (*F*_(3.80, 303.94)_ = 2.00, *p* = 0.10). Individuals with higher changes in the heart rate tended to show slightly *greater* sensitivity to instrumental contingency, as their response rate depended more strongly on programmed contingency. Thus, if any moderating effect of anxiety sensitivity exists, it goes in the opposite direction to what has been shown in individual difference research with stress and goal-directed control (e.g. Otto et al., 2013; Radenbach et al., 2015). Changes in the heart rate did not affect subjective causality ratings (*F*_(1, 80)_ = 0.08, *p* = 0.78). Similarly there was not a significant interaction between changes in the heart rate and programmed contingency (*F*_(3,29, 263.45)_ = 0.44, *p* = 0.74) in predicting subjective causality ratings.

### Anxiety induction and model-based planning (Experiment 2)

**Fig. 3.**
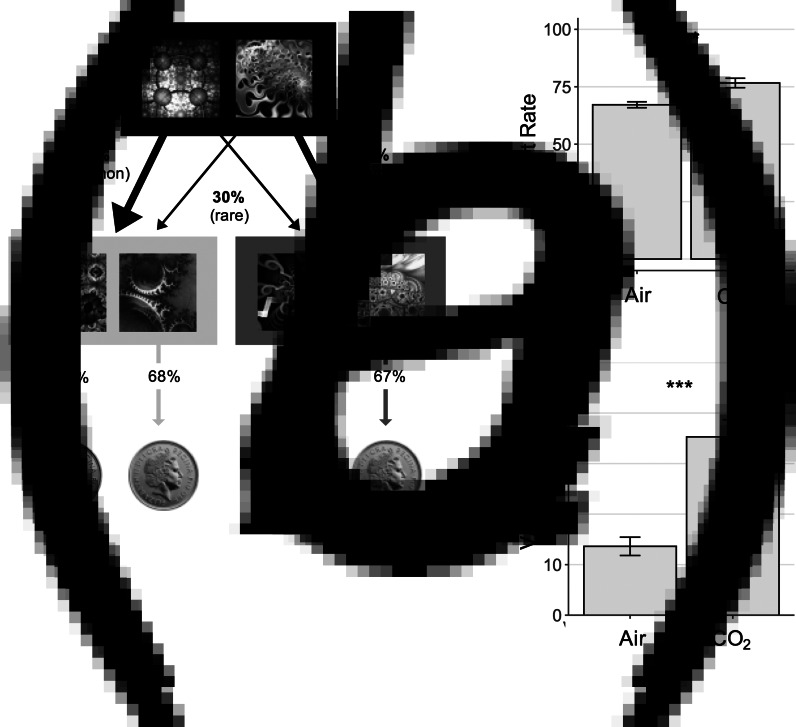
Experiment 2 Study Design – Model-Based Learning Task. (*a*) On each trial, subjects chose between two fractals, which probabilistically transition to either an orange or blue state (pictured here in greyscale) where they must make another choice. In this schematic, the fractal on the left had a 70% chance of transitioning to the blue state, what is called a ‘common’ transition, and a 30% chance of transitioning to the orange state, i.e. a ‘rare’ transition. In the second orange or blue state, subjects again chose between two fractals, each of which was associated with a probability of reward (a pound coin). Unlike the transition structure, these reward probabilities drifted slowly over time (0.25 < *p* < 0.75). This meant that subjects were required to dynamically track which of the fractals in the orange and blue states were currently best. The reward probabilities depicted (34%, 68%, 72% and 67%) refer to the probability of reward for each of the 4 options presented in an example trial at a certain point along the reward probability drifts. Model-based planning on this task is operationalised as the extent to which subjects' decision to repeat an action at the first stage, depend on (i) whether this action was rewarded on the previous trial and (ii) and whether the path from action to outcome was expected (‘common’). (*b*) Physiological response to anxiety induction. Heart rate was elevated significantly during the gas condition, *F*_(1,49)_ = 10.72, *p* = 0.002. Error bars represent s.e. (*c*) Psychological response to anxiety induction. Self-reported anxiety levels were also significantly elevated during the inhalation of gas compared with air, *F*_(1,49)_ = 57.47, *p* < 0.001. Error bars represent s.e. ***, *p* < 0.001.

We adopted a complementary approach to Experiment 1 to test if anxiety induction would affect goal-directed planning. We employed a ‘model-based’ learning task (Fig. 3) (Daw et al., 2005, 2011) in the context of a within-subjects design. The task is more commonly used in the literature and findings consistent with those in Experiment 1 would thus speak to the generalisability and validity of the findings. The within-subjects design overcomes the potential problem that individual differences in goal-directed control [e.g. associated with compulsiveness, IQ, age (Gillan et al., 2016)] may have hindered our ability to detect changes resulting from anxiety-induction in Experiment 1.

As in Experiment 1, the CO_2_ manipulation was effective in inducing anxiety in subjects (Fig. 3*b* and *c*), with a significant increase in self-reported anxiety *F*_(1,49)_ = 57.47, *p* < 0.001 and heart-rate, *F*_(1,49)_ = 10.72, *p* = 0.002. However, consistent with the results of Experiment 1, this did not alter goal-directed performance; CO_2_ had no effect on model-based planning (*β* = −0.03, s.e. = 0.04, *p* = 0.44). Bayes factor analysis indicated that there was moderate evidence in favour of the null model over the alternative model that included the acute anxiety manipulation (BF_01_ = 3.5).

**Fig. 4.**
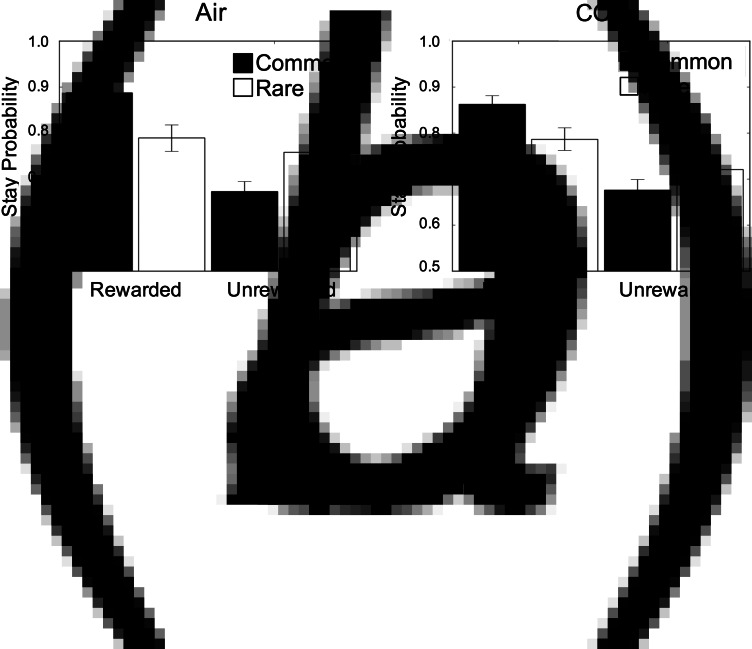
Results from Experiment 2. (*a*) Stay/switch behaviour for subjects in air condition as a function of whether or not the last trial was rewarded/unrewarded and followed a rare/common transition. Error bars represent s.e. (*b*) The same plot, showing the group average behaviour under CO_2_. In both plots, subjects showed the classic signatures of both model-based and model-free planning, indexed by a significant reward × transition interaction (*β* = 0.28, s.e. = 0.06, *p* < 0.001) and a main effect of reward (*β* = 0.55, s.e. = 0.08, *p* < 0.001). Error bars represent s.e.

The regression model overall fit subjects' behaviour as expected; ‘model-free’ behaviour was evident in the sample (*β* = 0.55, s.e. = 0.08, *p* < 0.001) which refers to how much subjects tend to repeat actions that were recently rewarded. Model-based learning was also overall significant (*β* = 0.28, s.e. = .06, *p* < 0.001), such that subjects took environmental contingency into account when deciding whether or not to repeat a rewarded choice. Finally, subjects showed an overall biased tendency to repeat choices from one trial to the next, regardless of reward or transition information (*β* = 1.59, s.e. = 0.12, *p* < 0.001). Much like model-based learning, there was no effect of anxiety on model-free learning (*β* = −0.02, SE = 0.03, *p* = 0.52), or action repetition (*β* = −0.08, s.e. = 0.04, *p* = 0.06; Fig. 4, online Supplementary Table S5). Although the latter approached significance such that subjects had a slight tendency to switch choices more while under CO_2_. These analyses were complemented with a full computational model (online Supplementary Material), with the only difference being that the effect of CO_2_ on choice switching was significant in this more comprehensive computational analysis (online Supplementary Table S8). Thus, it appears there may be a modest association between acute anxiety and an increased tendency to explore new options from trial to trial.

### Individual differences

Following the same logic as Experiment 1 – that individual differences in sensitivity to CO_2_ might be important in revealing the effect of stress on goal-directed behaviour and switching (Otto et al., 2013; Radenbach et al., 2015; Schwabe & Wolf, 2010) – we tested if the effects of CO_2_ on model-based planning might be detectible when we take into account how strongly subjects reacted to the CO_2_ manipulation. As we were not powered to construct a model with a 4-way interaction (which requires the inclusion of all subordinate interactions), we extracted individual coefficients for the effect of CO_2_ on model-based planning and switching and tested for correlation with subjects' change in self-reported anxiety and heart rate under CO_2_. There was no significant correlation between the effect of CO_2_ on model-based planning and change in anxiety, *r* = −0.20, *p* = 0.16, but there was a marginal association with a change in the heart rate under CO_2_, *r* = −0.29, *p* = 0.05. The analogous analysis from the computational model provided less support, where the correlation between change in self-reported anxiety was not significant, *r* = −0.20, *p* = 0.18, and nor was the correlation with a change in the heart rate, *r* = −0.15, *p* = 0.30. The Bayes factor indicated there was anecdotal evidence for the null with respect to the correlation between changes in self-reported anxiety and model-based planning (regression: BF_01_ = 1.3; computational model: BF_01_ = 1.4). For a change in the heart-rate, there was anecdotal evidence in favour of a relationship with the change in model-based planning in regression analysis (BF_10_ = 1.74), but anecdotal evidence in favour of the null from the computational analysis (BF_01_ = 1.9). Nonetheless, the direction of these trends, on the whole, suggested that those subjects whose model-based planning performance declined the most during CO_2_ may have also had the biggest psychological and physiological reaction to CO_2_. However, it is notable that (i) these results go in the *opposite* direction to those in Experiment 1 and (ii) if they exist, they are *very small*. To contextualise these findings in terms of the effect size, a sample of *N* = 258 would be needed for future studies to have 90% power to detect an association between change in anxiety and changes in model-based planning under CO_2_ using either the regression or computational model. For the heart-rate, *N* = 462 would be needed to have 90% power to detect an association with a change in the computational modelling parameterisation of model-based planning, and *N* = 119 to detect changes in the regression-defined model-model-based planning.

In contrast to model-based planning, there was a significant main effect of CO_2_ on switching. Though not the focus of the present study, we thus repeated the individual difference analysis for switching in an exploratory fashion. We found mixed evidence. There was an association with a change in self-report anxiety, where those individuals who were most anxious under CO_2_ tended to switch more under CO_2_ (regression: *r* = −0.43, *p* = 0.001; computational model: *r* = −0.29, *p* = 0.04). However, the same was not true for a change in the heart rate (regression: *r* = −0.13, *p* = 0.37, computational model: *r* = −0.09, *p* = 0.54). There was strong evidence that a change in self-reported anxiety correlated with a change in switching behaviour under CO_2_ in the regression (BF_10_ = 25.4), but only anecdotal evidence for this from the full computational model (BF_10_ = 2.17). For the heart rate, there was anecdotal evidence in favour of the *null* from both analyses (BF_01_ = 2.13; BF_01_ = 2.6).

### Real life anxiety (Experiment 3)

In two independent studies (Experiments 1 and 2) we found no effect of an acute anxiety induction on goal-directed planning. In a final experiment, we tested if anxiety in a real-life, more ecologically valid, setting might be necessary to reveal the hypothesised detrimental effect of anxiety on goal-directed behaviour. We tested 1413 subjects online using Amazon's Mechanical Turk on the model-based learning task described above. Findings relating to the association between compulsivity and model-based planning have been published elsewhere (Gillan et al., 2016), but in data not previously published, we enquired about whether subjects had a panic attack in the past week, which is known to induce a temporary state of acute anxiety. We chose to examine panic attacks, rather than using a questionnaire probing state anxiety, because state anxiety has an unacceptably high correlation with trait anxiety when measured in the absence of an acute stressor [e.g. *r* = 0.71 (Grös, Antony, Simms, & McCabe, 2007)]. As our prior work has already demonstrated that trait anxiety is not related to goal-directed planning (Gillan et al., 2016), we wanted to ensure that our measure of acute anxiety was not in large part confounded by trait anxiety. Measuring the occurrence of recent panic attacks is an attractive alternative (although not without limitation), because they represent an acute anxiety provoking event (Aronson & Logue, 1988) and as such is more comparable to our lab-based anxiety induction. Criteria for a panic attack were from item 1 of a validated instrument [PDSS (Shear et al., 1997)] and in brief required subjects to have experienced 4 of 17 symptoms (e.g. rapid or pounding heartbeat, feeling of choking, nausea, chills or hot flushes, fear of dying) and that the panic attack must have been a ‘sudden rush of fear or discomfort’, peaking within 10 min. Episodes like panic attacks that have fewer than four symptoms were defined as limited symptom attacks, but also contributed to subjects' score. Specifically, subjects indicated the frequency of panic or limited symptom attacks in the past week on item 1 of the PDSS and this served as our measure for subsequent analyses.

**Fig. 5.**
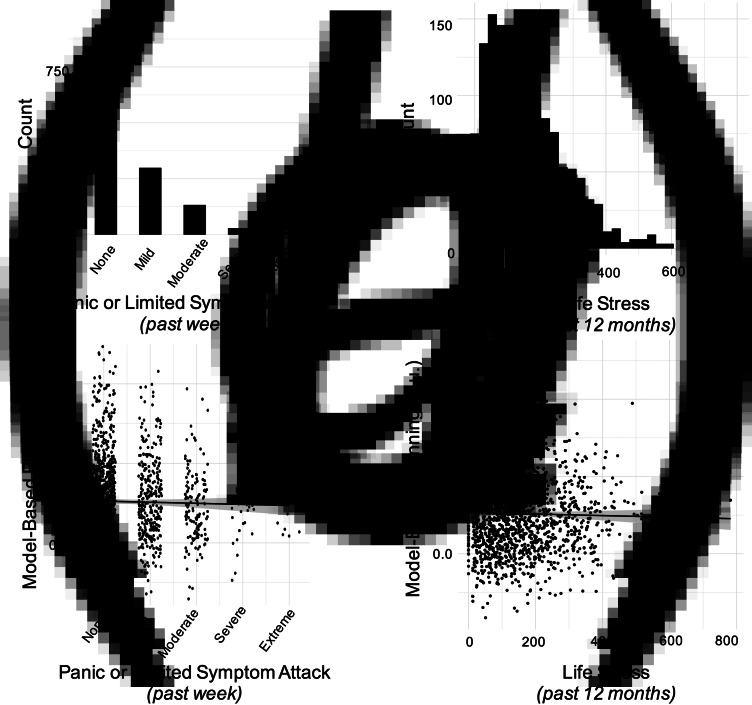
Results from Experiment 3. (*a*) Histogram displaying the number of individuals endorsing the various levels of frequency and severity of panic/limitd symptom attacks in the past week. Scores were coded as follows: none (‘no panic or limited symptom attacks’), mild (no full panic attacks and no more than 1 limited symptom attack/day), moderate (‘1 or 2 full panic attacks and/or multiple limited symptom attacks/day’), severe (severe: more than 2 full attacks but not more than 1/day on average) and extreme (‘full panic attacks occurred more than once a day, more days than not’). (*b*) Histogram displaying the distribution of life stress scores in the sample. (*c*) There was no association between model-based planning and the occurrence of panic attacks in the past week, after controlling for age, gender, IQ and compulsive symptomatology, *β* = −0.01, s.e. = 0.01, *p* = 0.33. The *Y*-axis displays residuals for model-based planning after these features are taken into account. (*d*) There was no association between model-based planning and life stress experienced over the past year, after controlling for age, gender, IQ and compulsive symptomatology, *β* = −0.01, s.e. = 0.01, *p* = 0.33. As above, the *Y*-axis displays residuals for model-based planning.

Consistent with other general population samples (Barrera, Wilson, & Norton, 2010), approximately a third (*N* = 474) of our online sample indicated they had experienced a panic or limited symptom attack in the past week (Fig. 5*a*). The frequency of panic attacks in the past week was correlated with reductions in model-based planning (*β* = −0.03, s.e. = 0.01, *p* = 0.012), but this did not survive controlling for ‘Compulsive Behaviour and Intrusive Thought’, a transdiagnostic psychiatric dimension that is negatively correlated with model-based planning (*β* = −0.04, s.e. = 0.01, *p* < 0.001); note this finding was previously published (Gillan et al., 2016). This compulsive factor presents a confound to interpretation of the panic results, because it is positively correlated with frequency of panic attacks (*r* = 0.42, *p* < 0.001). When compulsivity was accounted for, the effect of panic attacks on model-based planning was reduced to *β* = −0.01, s.e. = 0.01, *p* = 0.33 (Fig. 5*c*). Moreover, results from the more elaborate computational model showed that the effect of panic attacks on goal-directed planning approached zero and went in the opposite direction (*β* = 0.003, s.e. = 0.01, *p* = 0.81) after compulsivity was controlled for (online Supplementary Table S9).

We observed an association between the frequency of panic attacks and choice switching (*p* = 0.012), mirroring our causal result from Experiment 2. However, the effect of panic attacks on increased switching did not survive inclusion of compulsivity in the model for the one-trial-back regression (*p* = 0.23), or in the computational model (online Supplementary Table S9; *p* = 0.06).

Finally, we tested if life stress in the past year was associated with deficits in model-based planning. This was assessed using the Social Readjustment Scale (Holmes & Rahe, 1967), which presents an inventory of common stressful life events to participants and asks them to select those that applied to them in the previous 12 months (e.g. at the extreme end these include the death of a spouse or divorce) (Fig. 5*b*). Much like a recent panic attack, we found that life stress scores were linked to failures in model-based planning (*β* = −0.02, s.e. = 0.01, *p* = 0.04). However, as was the case for panic attacks, life stress was also correlated with the compulsive factor (*r* = 0.29, *p* < 0.001), and indeed the relationship to model-based planning did not survive inclusion of the compulsive factor in the analysis. Specifically, the effect of life stress on model-based planning was reduced to *β* = −0.01, s.e. = 0.01, *p* = 0.33 (Fig. 5*d*) in the regression analysis and *β* = −0.01, s.d. = 0.01, *p* = 0.24 in the full computational model (online Supplementary Table S10).

## Discussion

Across three independent studies, we found little or no evidence that anxiety has a detrimental effect on goal-directed planning. The first two experiments employed an extensively validated causal manipulation for inducing an acute state of anxiety, inhalation of air enriched with CO_2_ (Argyropoulos et al., 2002; Bailey et al., 2005). Using both between- and within-subject designs, and two well-validated tests for goal-directed behaviour, neither study found evidence that the causal manipulation had an effect on model-based planning. A third study took a correlational, but the larger scale (*N* = 1413), approach and tested if individuals who had panic/limited symptom attacks in the past week, which are associated with an increase in acute state anxiety (Aronson & Logue, 1988), had poorer goal-directed performance. Unlike most clinical studies, this design incorporated a comprehensive range of clinical assessments and could thus control for clinical confounds such as trait differences in compulsivity. While we found that those who experienced more panic attacks in the past week had poorer goal-directed planning, this did not survive controlling for compulsivity, a correlated trait that has been extensively studied in the content of goal-directed control failures. Together, these data contribute to a larger literature suggesting that trait (Gillan et al., 2016), and now state, anxiety do not have a clear detrimental effect on goal-directed planning in human subjects.

The most consistent cognitive changes that have been linked to trait anxiety are an increased attentional bias to threat or ‘hypervigilance’ (Mogg, Bradley, de Bono, & Painter, 1997) and the tendency to interpret ambiguous stimuli as threatening (Eysenck, Mogg, May, Richards, & Mathews, 1991). Results from studies using the 7.5% CO_2_ challenge closely mirror these findings – with the manipulation increasing alerting and orienting (Garner et al., 2012), threat processing (e.g. hypervigilance) (Garner et al., 2011) and negative interpretations of neutral events (Cooper et al., 2013), suggesting that 7.5% hypercapnic gas manipulation in the lab can mirror cognitive changes observed in association with anxiety. While the putative role that anxiety plays in more complex forms of decision-making is of broad interest (Paulus & Yu, 2012), there is a dearth of evidence suggesting it has effects that are not explained by increases in threat-sensitivity and vigilance. For example, while there is some evidence to suggest that clinically anxious individuals tend to make better long-term choices e.g. on the Iowa Gambling Task (IGT), this appears to result from a bias to avoid losses, which in the context of this task is confounded with the choice of ‘advantageous’ decks (Mueller, Nguyen, Ray, & Borkovec, 2010). Even this, however, has been inconsistently shown, with another study finding that high trait anxiety leads to *impaired* IGT performance (Miu, Heilman, & Houser, 2008). One potential explanation for inconsistent results in this area is that studies have been largely cross-sectional and correlational – something we overcame here by using causal manipulation of anxiety in studies 1 and 2.

Prior studies have suggested that, in the absence of a main effect of stress on goal-directed control, individual differences in sensitivity to the stressor itself may be important to consider (Otto et al., 2013; Radenbach et al., 2015). We repeated this general analytic approach here to facilitate comparison across studies, but the data were equivocal. There was no evidence that self-report anxiety or physiological sensitivity (i.e. heart rate) to the CO_2_ manipulation was associated with effects of the stressor on goal-directed behaviour in Experiment 1. One analysis of Experiment 2 data showed a marginal effect in the expected direction– diminished performance on the model-based learning task was observed in individuals that had the biggest change in heart rate under CO_2_. But notably, evidence was anecdotal and sometimes in favour of the null, depending on the analysis in question. Results varied depending on the measure of goal-directed planning was computational *v.* regression-based and whether the individual difference measure was self-report or physiological. More generally, it is difficult to interpret these effects in any causal framework given the absence of a main effect, such that these associations are driven, in part, by individuals who actually performed nominally *better* under CO_2_ (*N* = 22/50 in Experiment 2). Moreover, individual differences in sensitivity to CO_2_ is a somewhat problematic measure because it is itself a marker of mental health difficulties (Perna et al., 1996), presenting a confound to interpretation.

Although no previous studies have examined the effect of acute experimentally induced state anxiety on goal-directed control, several studies examined the impact of stress (Dias-Ferreira et al., 2009; Heller et al., 2018; Otto et al., 2013; Radenbach et al., 2015; Schwabe & Wolf, 2009, 2010) in healthy volunteers. Three studies found that stress-induced goal-directed deficits (Park et al., 2017; Schwabe & Wolf, 2009, 2010), mirroring findings in rodents following 21 days of unpredictable stress exposure (Dias-Ferreira et al., 2009). Three other studies, however, found no such effect (Heller et al., 2018; Otto et al., 2013; Radenbach et al., 2015). One key point of departure between studies reporting positive and negative results was the type of stressor used. Those that found significant effects used a socially-evaluated cold pressor test, and those that did not use either the cold pressor in isolation (Otto et al., 2013), or a social stress test in isolation (Heller et al., 2018; Radenbach et al., 2015). This distinction is important as the socially evaluated cold pressor test has been shown to induce a much stronger increase in cortisol, compared to cold pressor test alone (Schwabe, Haddad, & Schachinger, 2008), with the procedures otherwise eliciting similar cardiovascular and subjective stress responses. The notion that cortisol might mediate stress effects on goal-directed planning is supported by the observation that changes in cortisol were linked to deficits in performance in studies that failed to otherwise show a main effect of stress (Otto et al., 2013; Radenbach et al., 2015). In other words, the largest increases in cortisol were linked to the largest task deficits. This ties in with pharmacological evidence showing that decrements in goal-directed performance cannot be induced through noradrenergic manipulation alone; concurrent glucocorticoid stimulation is also necessary (although not sufficient) (Schwabe et al., 2010; Schwabe, Tegenthoff, Höffken, & Wolf, 2012). Differential involvement of cortisol might explain why acute stress appears to have an impact on goal-directed planning, but anxiety induction does not. While acute stress and anxiety induction result in similar cardiovascular effects (i.e. increases in heart rate and blood pressure) (Bailey et al., 2005; Schwabe et al., 2008) and noradrenergic activation (Allen, Kennedy, Cryan, Dinan, & Clarke, 2014; Bailey, Argyropoulos, Lightman, & Nutt, 2003), anxiety induction via 7.5% CO_2_ does not result in a reliable increase in cortisol (Oliveira, Chagas, Garcia, Crippa, & Zuardi, 2012; Woods et al., 1988). Hypercapnia causes more pronounced and specific increases in self-reported feelings of anxiousness, fear, panic and worry, which are reduced in response to common treatments for generalised anxiety, including anxiolytics (Bailey, Kendrick, Diaper, Potokar, & Nutt, 2007; Diaper et al., 2012). Therefore, it is possible that our results are specific to anxiety induction rather than stress *per se*.

The extent to which more chronic forms of real-life stress impair goal-directed control is an open question and has only been partially addressed in one prior study with a relatively small sample (*N* = 39) (Radenbach et al., 2015). Subjects with high self-reported chronic stress levels had a larger effect of acute stress on model-based planning performance, than their low stress counterparts (Radenbach et al., 2015). This might suggest that goal-directed learning is in some sense more fragile in individuals who have high levels of chronic life stress, but this is difficult to assess as the authors did not report any test for the direct association between life stress and model-based planning. We tested this using a large sample (*N* = 1413) and did not find evidence for an association, after controlling for compulsivity. This suggests that the impact of real-life stress on goal-directed planning, if it exists, is certainly less pronounced than folk wisdom suggests. That said, here we studied goal-directed behaviour, rather than habit expression *per se*, which represents a point of departure from some of the prior research e.g. in rodents (Dias-Ferreira et al., 2009). Further work is needed in this direction as it is possible that any effect of anxiety is on habit expression, and not goal-directed control.

In Experiments 2 and 3, there was a suggestion that subjects' tendency to switch their choices from one trial to the next was increased following anxiety induction and the recent occurrence of a panic attack, respectively. These findings were not hypothesised and effect sizes were somewhat inconsistent across analysis methods, but given their consistency with a prior independent study (Radenbach et al., 2015), they warrant brief discussion. One possibility is that this increase in choice switching might reflect the enhanced uncertainty characteristic of anxious states (Grupe & Nitschke, 2013) and could arise as a result of activation of the noradrenergic system (Redmond & Huang, 1979; Yu & Dayan, 2005). Evidence for this comes from work suggesting that tonic noradrenaline release is linked to an increase in task irrelevant processing and a tendency to favour exploration over exploitation (Aston-Jones & Cohen, 2005), characterised by some as a network ‘reset’ (Bouret & Sara, 2005). This interpretation is limited by the absence of data on cortisol and noradrenaline response and the exploratory nature of the findings. Future research will be needed to test this more directly, using a cognitive test designed to explicitly separate exploration and exploitation.

This study had limitations. Firstly, null results are difficult to draw firm conclusions from. However, the findings of Experiment 3, which benefit from the inclusion of a previously published clinical effect size comparator (the effect of compulsivity on model-based planning), help to place these null findings into a meaningful context. It is unlikely that our manipulation was not strong enough to induce a robust anxiogenic effect because previous studies have demonstrated that the 7.5% CO_2_ manipulation is powerful enough to elicit robust effects on behavioural performance relating to threat sensitivity and hyper-vigilance (Cooper et al., 2013; Garner et al., 2011, 2012), in addition to its well-documented physiological and psychological effects (Bailey et al., 2005, 2007). The magnitude of self-report and physiological changes in the present study was on-par with those observed in prior studies (Cooper et al., 2013; Garner et al., 2011, 2012). Finally, Bayesian analyses detail the extent to which evidence was in favour of the null, and this was in most cases in the ‘very strong’ range. A second limitation is that using panic attacks to measure ‘real world’ state anxiety is an imperfect methodology. Although panic attacks are associated with an increase in state anxiety (Aronson & Logue, 1988), they are also associated with, and defined by, a much broader cascade of physical symptoms than the experience of state anxiety. However, this approach has two advantages over measuring self-reported state anxiety [e.g. using the STAI-state scale (Spielberger, Gorsuch, Lushene, Vagg, & Jacobs, 1983)]. First, in the absence of an acute event (anxiety trigger), trait and state anxiety scores tend to be highly correlated [e.g. *r* = 0.71 (Grös et al., 2007)] and the STAI-scale is thus thought to be more reflective of trait than state anxiety. Second, leveraging naturally occurring panic attacks allowed us to mirror the acute and sudden onset of anxiety that our lab-based procedure achieved.

## Conclusions

Experimentally induced state anxiety failed to produce deficits in goal-directed behaviour as measured via two independent experiments using two well-validated probes. Such lack of effect was also observed in a more ecologically valid set-up, where we used recent panic attacks as a proxy for acute anxiety. While modest decreases in goal-directed planning were seen in individuals who had recent panic attacks in the past year, these effects did not survive when controlling for compulsivity. The same was true of the occurrence of major life stressors in the past year. In terms of clinical implications, these data suggest that state anxiety has little *specific* effect on goal-directed control, in contrast for example to compulsivity, which research has shown has a consistent association. This distinction may have important implications for the development of differential treatment approaches for patients who present with the same diagnosis, for example of OCD, but differ substantially in their levels of anxiety *v.* compulsivity. Dimensional approaches that seek to distinguish these dimensions and target them individually present a new frontier for psychiatry research aiming to develop more personalised treatment approaches. For future research studies more generally, these data highlight the necessity of using positive clinical control measures and causal manipulations to ascertain robust and specific associations given a deeply complex and highly inter-correlated mental health landscape.

## Data Availability

The datasets generated during and/or analysed during the current study are freely available on the Open Science Framework (https://osf.io/w4yfp/).

## Supplementary material

For supplementary material accompanying this paper visit https://doi.org/10.1017/S0033291720000203. click here to view supplementary material

## Code availability statement

Code to reproduce results is freely available on the Open Science Framework (https://osf.io/w4yfp/).
