# Supplementary material for: Experimentally induced and real-world anxiety have no demonstrable effect on goal-directed behaviour
Source: Psychol Med. 2020 Mar 2;51(9):1467–78. doi: 10.1017/S0033291720000203 (PMC8311820; doi:10.1017/S0033291720000203)
Supplement: Supplementary file 1 [file S0033291720000203sup001.docx]

**Supplementary Materials for:**

**Experimentally-induced and real-world anxiety have no demonstrable effect on goal-directed behaviour.**

*Gillan CM^1^, *Vaghi MM^2,3^, Hezemans FH^2^, van Ghesel Grothe S^4^, Dafflon J^5^, Brühl AB^6^, Savulich G^2^, & Robbins TW^2^

^1^ Trinity College Dublin, Dublin 2, Ireland

^2^ University of Cambridge, Cambridge, United Kingdom

^3^ Present address: Max Planck UCL Centre for Computational Psychiatry and Ageing Research, 10-12 Russell Square, London, United Kingdom

^4^ University of Amsterdam, Netherlands

^5^ Kings College London, London, United Kingdom

^6^ University Hospital of Psychiatry Zurich, Switzerland

*Denotes equal first author contribution

**Experiment 1**

**Subjects**. Recruitment and experimental procedures were approved by the Ethics Committee of the University of Cambridge, School of the Biological Sciences. For subjects included in the experiment, exclusion criteria were screened by a structured telephone interview and were as follows: current or past diagnosis of cardiovascular disease, respiratory disease, thyroid disease, or diabetes; lifetime history of DSM-VI Axis I disorders (Mini International Neuropsychiatric Interview: MINI (Sheehan *et al.*, 1998)); having a first-degree relative diagnosed with panic disorder; (history of) migraine or epilepsy; pregnancy; excessive weekly consumption of alcohol (28 units for males, 21 units for females), excessive daily consumption of caffeine (more than 8 caffeinated drinks per day); current (illegal) drug use; recent history of smoking on a daily basis. Participants were free of regular medication intake, with the exception of oral contraceptives. Invited participants were asked to abstain from alcohol consumption 24 hours prior to the experiment, as well as caffeinated drinks from the midnight before the experiment. Sample size was determined based on a previous study (Schwabe and Wolf, 2010) that found a between-subjects effect of stress on habitual performance with partial eta squared = .07. 88 subjects were required to reproduce an effect of this size with 80% power. Participants were reimbursed for their time and informed consent was obtained prior to participation.

**Contingency degradation manipulation.** Participants were tested on the ability to detect action-outcome instrumental contingency via the experimental manipulation of contingency degradation. Our index of contingency was the standard ΔP measure indexing the action-outcome instrumental relationship (Dickinson and Balleine, 1994). ΔP was the difference between the conditional probability of outcome given an action [P(O|A)], i.e., the probability of response-contingent outcome; and the probability of receiving an outcome given the absence of an action [P(O|-A)], i.e., the probability of a non-contingent outcome, such that ΔP = P(O|A) - P(O|-A). By increasing non-contingent outcomes, the contingency (i.e., the causal action-outcome association) is degraded. Under these circumstances, individuals who are making decisions in a goal-directed manner should stop or reduce responding in line with the reduction in instrumental contingency.

**Contingency degradation paradigm.** In a free-operant, self-paced procedure, a white triangle on the screen signalled that the participant was free to press, or not to press, the space bar. On each response, the triangle turned yellow until the end of the *a priori* specified bin to signal that a response has been recorded and to prevent multiple responses within the same 1-second bin. When a reward was delivered, following a key press or not, an image of a 25 pence coin was shown at the end of the bin for 500 milliseconds with the text “Reward, you win!” and a sound. If no outcome was delivered, no feedback was given and the next bin started. Each participant completed 8 blocks where ΔP was systematically varied (Figure 1B). A running total of money earned within the block was displayed in the corner of the screen and reset to 0 at the beginning of each block. Causality judgments regarding the relationship between pressing the key and receiving the reward were collected at the end of each block. Each block included 140 un-signalled bins, each lasting 1 second. If a response occurred during a given bin, the outcome was delivered with probability P(O|A) defined a priori for that block; if no response occurred, the outcome was delivered with probability P(O|-A) defined a priori for that block. Only the first space-bar press within the bin had any programmed consequences. By varying P(O|A) and P(O|-A), different levels of instrumental contingency were established in each block. In the first 2 blocks, all participants inhaled normal air and the associated programmed contingencies were always presented in the same order (high contingency 0.6, followed by degradation of the contingency to 0.3), providing an implicit training phase. The remaining blocks (test phase) were presented according to a Latin square design for participants in each of the two experimental groups.

Prior to the experiment, the instructions informed the participants that they could earn 25 pence whilst pressing the space bar on a keyboard, and that they were free to press the key as often as they liked. They were further instructed that the relationship between pressing the space bar and receiving the 25p reward would vary during the experiment, and that pressing the space bar might earn a reward, a reward might also arrive on its own, or pressing the space bar might prevent a reward from arriving. Lastly, they were informed that occasionally they would be asked to rate the degree to which pressing the space bar caused the occurrence of the reward.

**Table S1: Programmed contingency, experienced contingency, response rate, and causality judgement for each experimental block.**

|  |  |  | **Programmed**  **contingency** | | | **Experienced contingency** | | **Response**  **Rate** | | **Causality judgment** | |
| --- | --- | --- | --- | --- | --- | --- | --- | --- | --- | --- | --- |
|  | **Block** | | **P(O\|A)** | **P(O\|-A)** | **ΔP** | **Air** | **CO_2_** | **Air** | **CO_2_** | **Air** | **CO_2_** |
| **Fixed Order** | 1 | | 0.60 | 0.00 | 0.60 | 0.61 | 0.60 | 0.54 | 0.57 | 58.79 | 63.19 |
|  | 2 | | 0.60 | 0.30 | 0.30 | 0.36 | 0.35 | 0.42 | 0.46 | 40.15 | 43.84 |
| **Shuffled in a Latin square** | 3 | | 0.60 | 0.00 | 0.60 | 0.60 | 0.59 | 0.64 | 0.59 | 56.29 | 58.97 |
|  | 4 | | 0.60 | 0.10 | 0.50 | 0.52 | 0.51 | 0.55 | 0.55 | 47.53 | 51.61 |
|  | 5 | | 0.60 | 0.30 | 0.30 | 0.30 | 0.31 | 0.50 | 0.42 | 42.68 | 40.53 |
|  | 6 | | 0.60 | 0.40 | 0.20 | 0.21 | 0.19 | 0.40 | 0.45 | 38.65 | 40.22 |
|  | 7 | | 0.60 | 0.50 | 0.10 | 0.07 | 0.06 | 0.43 | 0.40 | 33.66 | 36.87 |
|  | 8 | | 0.60 | 0.60 | 0.0 | -0.02 | -0.03 | 0.39 | 0.33 | 28.27 | 28.10 |

**P(O|A), probability of the outcome given the action; P(O|-A), probability of the outcome in the absence of the action; ΔP =contingency. Dependent variables are given as mean. Blocks 1-2 were presented in a fixed order; Block 4-8 were presented according to a Latin square design. Programmed contingency refers to the a priori experimentally programmed contingency resulting from the a priori programmed conditional probabilities. Experienced contingency was computed on the basis of experienced event frequencies.**

**Psychological and physiological response to stress.** Psychological and physiological measures confirmed that participants in the CO_2_ condition experienced greater anxiety and stress than those assigned to the Air condition (Figure 1B and 1D). Group means and standard deviations are presented in Table S2 and S3, respectively. Subjective ratings of negative affect increased under CO_2_; there were significant group by time interaction effects (all *p* < .001) for the API, the PANAS negative affect subscale, as well as the “fearful” and “anxious” visual analogue scales (Figure 1D). The results for positive affect were mixed; happiness decreased under CO_2_ (*p* = .004), but there was no significant difference for the (more extensive) PANAS positive affect subscale (*p* = .84). In terms of autonomic measures of arousal, there were significant group by time interaction effects (all *p* < .001) for heart rate, systolic blood pressure and diastolic blood pressure. As shown in Figure 1B, heart rate and blood pressure significantly increased under inhalation of CO_2_ compared to normal air.

**Relationship between response rate and causality judgments.** As goal-directed control involves the implementation of contingency knowledge into flexible action, we lastly tested the extent to which causality judgments predicted response rate, and whether that might be affected by CO_2_-induced anxiety. Overall, response rate was linearly predicted by causality ratings (*F*_(1, 96.44)_ = 78.18, *p* < .001), but the slope of this relationship was not significantly different between groups (group by causality judgement interaction effect: *F*_(1, 96.44)_ = 0.05, *p* = .83) (Figure 2B). This analysis thus indicated that the linear relationship between subjectively detected instrumental contingency and response rate remained intact in face of an acute anxiety induction. To examine the relative evidence for the null we used Bayes analysis from the ‘bmrs’ package in R. For this specific model (i.e., mixed models) there is no “default” and we had to set our own priors. We standardized the data and used relatively wide (i.e., ‘weakly informative’) priors, following recommendations from the ‘brms’ package documentation (Bürkner ,2017). Specifically, we used normal priors with mean=0 and standard deviation=10 for the fixed effect parameters; half student-t priors with degrees of freedom=3, location=0 and scale=10 for the standard deviation of the random effects; and an LKJ prior with shape=1 for the correlation between random effects. The null model (including only causality judgements) was very strongly preferred over the alternative model with fixed effect of anxiety and anxiety by causality judgement interaction (BF_01_ = 5882.35).

**Table S2: Means and standard deviations for positive and negative affect by group and time**

|  | **Pretest** | | **Test** | | **Post-test** | |
| --- | --- | --- | --- | --- | --- | --- |
|  | **Air** | **CO_2_** | **Air** | **CO_2_** | **Air** | **CO_2_** |
| **API** | 1.80 ± 2.00 | 2.88 ± 3.28 | 2.98 ± 3.69 | 16.5 ± 8.17 | 3.10 ± 3.99 | 4.35 ± 6.05 |
| **VAS** |  |  |  |  |  |  |
| Anxious | 17.49± 16.95 | 18.19± 18.10 | 11.39± 11.77 | 42.34± 27.08 | 13.22± 14.05 | 16.83± 19.82 |
| Fearful | 14.48± 19.15 | 12.88± 16.22 | 10.23± 15.12 | 35.59± 27.52 | 11.84± 17.76 | 14.16± 19.70 |
| Happy | 63.23± 18.20 | 53.66± 19.96 | 54.26± 19.98 | 34.11± 19.62 | 59.05± 21.93 | 52.70± 22.69 |
| **PANAS** |  |  |  |  |  |  |
| Negative | 12.67 ± 3.27 | 12.71 ± 3.23 | 11.41 ± 2.24 | 17.28 ± 6.42 | 12.19 ± 3.48 | 12.65 ± 4.62 |
| Positive | 27.60 ± 7.76 | 26.95 ± 8.35 | 22.14 ± 8.30 | 20.67 ± 7.92 | 24.43 ± 9.29 | 23.47 ± 8.64 |

**API, Acute Panic Inventory; VAS, Visual Analogue Scale; PANAS, Positive and Negative Affective Scale. Data show mean and standard deviation.**

**Table S3: Means and standard deviations for autonomic arousal by group and time**

|  | **Pretest** | | **Test** | | **Post-test** | |
| --- | --- | --- | --- | --- | --- | --- |
|  | **Air** | **CO_2_** | **Air** | **CO_2_** | **Air** | **CO_2_** |
| **HR** | 70.0± 10.4 | 70.1± 10.5 | 70.3± 10.0 | 86.0± 15.8 | 68.1± 11.1 | 68.0± 13.2 |
| **BP systolic** | 116.0±13.6 | 117.7±16.3 | 115.0±12.3 | 140.4±22.8 | 115.2±20.1 | 123.3±16.7 |
| **BP diastolic** | 69.2± 8.2 | 71.6± 10.4 | 70.5 ± 9.9 | 83.7± 15.8 | 72.2 ± 9.7 | 78.5± 11.7 |

**HR, rate rate; BP, blood pressure. Data show mean and standard deviation.**

**Figure S1**


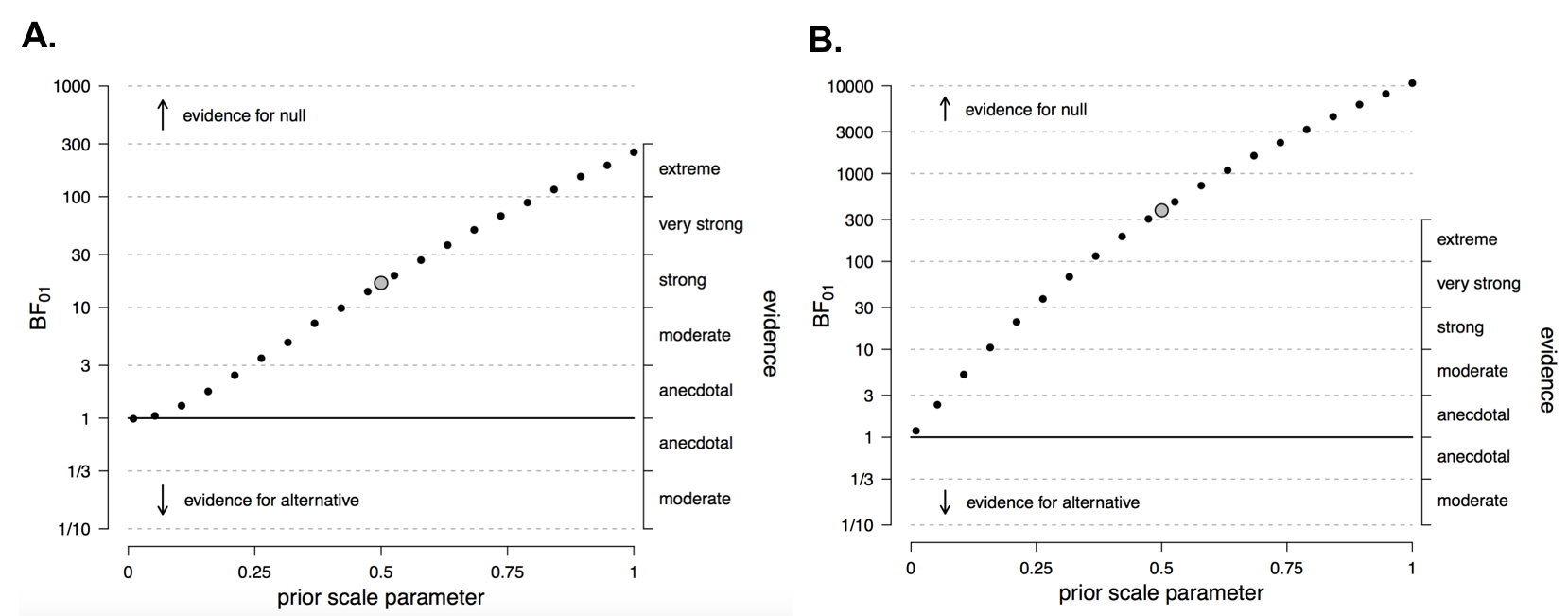


**Figure S1**. Bayes Factor as a function of the scale parameter of the Cauchy prior for the fixed effects under the alternative hypothesis. The grey dot indicates the result from the default prior (scale parameter = 0.5). As the scale parameter increases (i.e. the prior becomes wider), the Bayes Factor increasingly favors the null hypothesis. Even under the prior setting that most favors the alternative hypothesis (i.e. scale parameter close to zero), the Bayes Factor remains in favor of the null hypothesis, both in the case of response rate (**A**) and subjective causality ratings (**B**).

**Supplementary Information for Experiment 2**

**Subjects.** Four subjects aborted the experiment and data were lost from an additional 3. Because of the nature of the analyses, subjects were excluded if their stay/switch behaviour showed such little variation as to preclude a hierarchical model-fit (choosing same response >90% of trials, N=3) or conversely, deviated substantially (>3 SDs) from the mean in the opposite direction (N=1). Sample size was determined based on a previous study (Schwabe and Wolf, 2010) that found a between-subjects effect of stress on habitual performance with partial eta squared = .07. Using a within-subject design, and assuming a .5 correlation across within-subject conditions, we determined that 47 subjects were required to reproduce an effect of this size with 95% power. The study was approved by the same ethics committee as Experiment 1. Participants were reimbursed for their time and informed consent was obtained prior participation.

**Reinforcement learning task.** On each trial, participants were presented with a choice between two fractals, each of which commonly (70%; see Figure 3A) led to a particular second state displaying another two fractals. These second-state fractals each had some probability (between .25 and .75) of being rewarded with a pound coin. On 30 % of trials (“rare” transition trials; Figure 3A), choices uncharacteristically led to the other state. A purely model-free learner makes choices irrespective of these contingencies (i.e. which action is most strongly linked to which second stage state), and instead focuses on repeating actions that were followed by reward. A model-based strategy, in contrast, is characterized by sensitivity to both reward and the transition structure (contingency) within the task. This means that when a stage 1 action is ultimately rewarded at the end of a trial, a model-based learner will repeat that stage 1 action again, only if the path to reward they took was likely (i.e. involved a common transition). If the path they took to reward was unlikely (involving a rare transition), a model-based subject switches their stage 1 action to promote their chances of returning to that valuable second stage state. The chances that a second stage fractal would be rewarded drifted slowly over time, such that in order to perform optimally, subjects needed to update action preferences dynamically throughout the task.

Before starting the task, participants completed a training session, which comprised written instructions, the viewing of 20 trials demonstrating the probabilistic association between the second stage fractals and coin rewards, and completion of 20 trials of active practice with the probabilistic transition structure of the task. Subjects were then tested for their comprehension of the task with a short quiz (Gillan *et al.*, 2016a) and if they answered any questions incorrectly, these comprehension issues were clarified on-screen. The task consisted of 200 trials in which participants had 2.5 s in which to make a response using the left and right keys following presentation of the first-state choice. If no response was made on time, “no response” were presented on the screen, and the next trial started. If a choice was made, the selected fractal moved to the top centre of the screen and shrunk in size. A new, second-state fractal appeared in the centre of the screen and was followed by an image of a pound coin or a zero. Subjects completed two counterbalanced versions of the task, with different fractal stimuli and reward drifts. Model-based planning has been previously shown to correlate with sensitivity to outcome devaluation (Gillan *et al.*, 2015), OCD diagnosis (Voon *et al.*, 2014), symptoms (Gillan *et al.*, 2016a), and has been successfully modified using pharmacological manipulations (Wunderlich, Smittenaar and Dolan, 2012; Worbe *et al.*, 2015). As such, it represents an established test of goal-directed planning.

**Psychological and physiological response to stress.** Under acute CO_2_ administration, subjects were more anxious, fearful, and less happy (all *p*<.001, Table 2). Subjects’ scores on the acute panic index (API) also increased (*p*<.001) under CO_2_, as did their heart-rate (*p*<.001) and blood pressure (*p*<.001). Subjects also reported more negative affect (*p*<.001) and less positive affect (*p*=.029) on the PANAS.

**Table S4. Self-report within-subject changes associated with acute CO_2_ administration**

|  | **Air** | **CO_2_** | **F** | ***p*** |
| --- | --- | --- | --- | --- |
|  | *Mean (SD)* | *Mean (SD)* |  |  |
| **API** | 3.6 (4.0) | 13.4 (9.0) | 64.86 | < .001*** |
| **PANAS PA** | 24.6 (8.4) | 22.4 (8.9) | 5.16 | = .03* |
| **PANAS NA** | 11.5 (2.7) | 15.9 (5.1) | 55.1 | < .001*** |
| **VAS anxious** | 13.6 (12.6) | 35.3 (24.0) | 57.47 | < .001*** |
| **VAS fearful** | 9.4 (9.8) | 25.6 (24.1) | 35.44 | < .001*** |
| **VAS happy** | 51.5 (20.7) | 41.6 (22.2) | 15.72 | < .001*** |
| **BP-systolic^** | 114.8 (15.2) | 133.9 (22.2) | 74.17 | < .001*** |
| **BP-diastolic^** | 73.1(12.0) | 81.1(15.3) | 15.7 | < .001*** |
| **HR^** | 67.2 (9.0) | 76.6 (14.4) | 25.28 | <.001*** |
|  |  |  |  |  |

**SD= standard deviation; API = acute panic index; PANAS= positive and negative affect schedule; PA= positive affect; NA= negative affect’ VAS= visual analogue scale; BP= blood pressure; HR= heart rate.**

**^BP data were missing for 1 subject and HR was missing for 2 subjects.**

**** p<.05; **p<.01, ***p<.001***

**Detailed Results for Model-Based Task.** The regression model fit subjects’ behavior as expected, based on the prior literature; there was a significant main effect of Reward (β =.55, SE=.08, *p*<.001) and a significant Reward x Transition interaction (β=.28, SE=.06, *p*<.001), providing evidence that, overall, subjects’ choices showed signatures of both model-free and model-based processes. The intercept was significant; subjects had an overall tendency to repeat choices from one trial to the next, β=1.59, SE=.12, *p*<.001) (Table S5). Importantly, CO_2_ had no effect on subjects’ tendency to exhibit model-based (β=-0.03, SE=0.04, *p*=.44) or model-free (β=-0.02, SE=0.03, *p*=.52) behavior. There was a non-significant trend for subjects to switch more under CO_2_ (main effect of CO_2_ condition, β=-0.08, SE=0.04, *p*=.060; Table S5).

**Table S5. Results from regression model for Experiment 1**

|  |  | | |
| --- | --- | --- | --- |
| **Coefficient** | **β (SE)** | ***z*-value** | ***p*-value** |
| (Intercept) | 1.59(0.12) | 12.88 | <.001 *** |
| Reward | 0.55(0.08) | 6.74 | <.001 *** |
| Transition | 0.08(0.04) | 1.96 | 0.05 * |
| CO_2_ | -0.08(0.04) | -1.85 | 0.06 |
| Reward:Transition | 0.28(0.06) | 4.48 | <.001 *** |
| Reward:CO_2_ | -0.02(0.03) | -0.65 | 0.52 |
| Transition:CO_2_ | 0.04(0.03) | 1.48 | 0.14 |
| Reward:Transition:CO_2_ | -0.03(0.04) | -0.76 | 0.44 |
|  |  |  |  |

****p*<.05 ** *p*<.01 ****p*<.001**

**SE=standard error**

**Computational Modeling Method**

*Reinforcement Learning (RL) Model*

We used a reinforcement-learning (RL) model based on a hybrid of model-free Q_MF_(s_A_, a) and model-based Q_MB_(s_A_, a), as utilized in previous studies (Sharp *et al.*, 2016; Daw *et al.*, 2011). This model consists of separate model-based and model-free subcomponents, both of which estimate a state-action value function, which maps each possible action to its expected future reward. On trial t, we denote the first-stage state (always s_A_) by s_1,t_, the second-stage states by s_2,t_, the chosen first-stage action by a_t_, and the second-stage rewards as r_t_ .

For the model-free algorithm, we used temporal difference (TD) learning (Rummery and Niranjan, 1994), which updates the value for the visited state-action pair at s_1,t_ according to: $Q_{MF}\left( s_{1,t},a_{t} \right)=Q_{MF}\left( s_{1,t},a_{t} \right)+\alpha\delta_{1,t}$

where α is a learning rate parameter and$\delta_{1,t}$ is the reward prediction error (RPE) at state 1, trial t:$\delta_{1,t}=Q_{MF}\left( s_{2,t} \right) {- Q}_{MF}\left( s_{1,t},a_{t} \right)$

The RPE is based on the second-stage value, $Q_{MF}\left( S_{2,t} \right).$Second-stage values are themselves updated according to: $Q_{MF}\left( s_{2,t} \right)=Q_{MF}\left( s_{2,t} \right)+\alpha\delta_{2,t}$

where the RPE at the second stage state, trial t $(\delta_{2,t})$ is determined by whether or not the trial was rewarded, $r_{t}$:$\delta_{2,t}=r_{t} {- Q}_{MF}\left( s_{2,t} \right)$

The model assumes that the eligibility trace =1 for all subjects (Sharp *et al.*, 2016), thus propagating second-stage reward information to the first-stage values. At the end of each trial, we decayed the Q values for all of the non-selected actions by multiplying them by 1 − α (Lau and Glimcher, 2005; Ito and Doya, 2009).

The model-based RL algorithm works by learning the transition structure of the task (the state most often visited previously after each top-stage choice) and immediate reward values for each second stage state, then computing cumulative state-action values by iterative expectation over these. At the second stage (where immediate rewards were offered), the problem of learning immediate rewards is equivalent to that for TD above, because $Q_{MF}\left( s_{2t} \right)$ is just an estimate of the immediate reward r_t_; with no further stages to anticipate, and the SARSA learning rule reduces to a delta rule for predicting the immediate reward. Thus, the two approaches coincide at the second stage, and we define Q_MB_ = Q_MF_ at those states. Critically, the top level model-based values are defined from both the transition and reward estimates using the Bellman Equation (Bellman, 1957):

$$Q_{MB}\left( s_{A}{,a}_{A_{j}} \right)={P\left( s_{B}|s_{A}{,a}_{j} \right) Q}_{MF}\left( s_{B} \right)+{P\left( s_{C}|s_{A}{,a}_{j} \right) Q}_{MF}\left( s_{C} \right)$$

where we have assumed these are recomputed on each trial from the current estimates of the transition probabilities and rewards. To connect the model-based and model-free values to choices, we use a softmax choice rule, which assigns a probability to each action based on a weighted sum of model-based and model free values (Otto *et al.*, 2013). The probability of each choice at the first stage is calculated, accordingly, as

$$P\left( a_{t}= a|s_{1,t} \right)=\frac{{exp[\beta_{MB}\cdot Q}_{MB}\left( s_{1,t},a \right)+\beta_{MF}\cdot Q_{MF}\left( s_{1,t},a \right)+p\cdot rep(a)]}{\Sigma_{a^{'}} {exp[\beta_{MB}\cdot Q}_{MB}\left( s_{1,t},a' \right)+\beta_{MF}\cdot Q_{MF}\left( s_{1,t},a' \right)+p\cdot rep(a')]}$$

The indicator function rep($a$) is defined as 1 if $a$ is the same one as was chosen on the previous trial, zero otherwise. Together with the “stickiness” parameter p, this captures first-order perseveration (p > 0) or switching (p < 0) in the first- stage choices (Lau and Glimcher, 2005). Second-stage choices are modeled with only a single value term$Q_{MF}\left( s_{1,t},a \right)$ with its an inverse temperature β and no stickiness parameter.

This model was embedded within a multi-level random effects model of the population variation in its parameters to estimate it for all subjects simultaneously and to estimate the effect of condition on these parameters, i.e. CO_2_ (on/off). This was done identically to Sharp and colleagues (2016), in that the within-subjects effect of CO_2_ is a subject-specific latent variable with its own population-level mean and variance, which are themselves inferred. All of the parameters of the model were taken as random effects, instantiated separately for each subject *s* from a common group level distribution. We estimated the parameters of the group level distributions using uninformative priors: for all parameters, the prior means and SDs were the heavy-tailed *Cauchy*(0,2), with the exception of α, where we selected narrower prior distributions so that the sigmoid-transformed parameters were roughly uniform in [0,1] a priori; prior mean and SD were *Normal*(0,1).

We estimated the joint distribution of the parameters of the model, conditional on all subjects’ observed choices and rewards. For this, we used Markov Chain Monte Carlo (MCMC) techniques (specifically the No-U-Turn variant of Hamiltonian Monte Carlo) as implemented in the Stan modeling language (v2.5, 2014). Given a probabilistic generative model (the above equations) and a subset of observed variables, MCMC techniques provide samples from the conditional joint distribution over the remaining latent variables. We ran four chains of 4,000 samples each, discarding the first 2,000 samples of each chain for burn-in. We examined the time-series plots of the chains visually for convergence and also computed Gelman and Rubin’s (1992) potential scale reduction factors. For this, large values indicate convergence problems, whereas values near 1 are consistent with convergence. We ensured that these diagnostics were less than 1.02 for all variables.

**Computational Modeling Results for Experiment 2**

Using the complementary computational analysis detailed above, we estimated learning rates and choice stochasticity, in addition to model-based, model-free and exploratory behaviour. This allowed us to test if changes in learning rates and/or choice randomness might explain our findings of increased exploration under CO2. Consistent with the one-trial back regression analysis, CO_2_ had a significant effect on stay/switch behaviour only, such that subjects were more likely to switch to a new action under acute CO_2_ (Table S7). This does not correspond to more randomness in choice, which is captured by the stochasticity parameter.

**Table S6. Group-level estimates of the effect of CO_2_(on/off) on each free parameter in the computational model.**

| **Influence of CO_2_ (ON/OFF) on Model Parameter Estimates** | | | | | |
| --- | --- | --- | --- | --- | --- |
| **Upper 95%**  **Median**  **Lower 95%** | **α**$\mathbf{CO}_{\boldsymbol{2}}$ | ***p***$\mathbf{CO}_{\boldsymbol{2}}$ | $\boldsymbol{mb}\mathbf{CO}_{\boldsymbol{2}}$ | $\boldsymbol{m}\mathbf{fCO}_{\boldsymbol{2}}$ | $\boldsymbol{beta}\boldsymbol{2}\mathbf{CO}_{\boldsymbol{2}}$ |
|  | 0.29 | **-0.04** | 0.07 | 0.23 | 0.12 |
|  | -0.21 | **-0.16** | -0.05 | 0.08 | -0.11 |
|  | -0.60 | **-0.27** | -0.17 | -0.06 | -0.35 |

***α* = learning rate; *p* = perseveration; *mb* = model-based; *mf* = model-free; *beta2* = choice stochasticity.**

**For the effect of CO_2_ on each parameter, the median posterior estimate is given, together with the 95% confidence intervals. Only the slope of *p*CO_2_ (i.e. the effect of CO_2_ on perseveration) is significantly different from zero, such that subjects were more likely to switch choices from trial to trial (i.e. perseverate less) under CO_2_.**

**Experiment 3Subjects.** Participants were paid a base rate of $2.50, in addition to a bonus based on their earnings during the reinforcement-learning task (M=$0.54, SD=0.04). This study was approved by the New York University Committee on Activities Involving Human Subjects. These participants are the same as those in a previously published article (Gillan *et al.*, 2016a). Participants provided their consent online after reading the study information in agreement with the requirements of the relevant research committee. Sample size was determined using pilot data N=548 from a prior study (Gillan *et al.*, 2016a) suggesting that to achieve 80-90% power to detect an association between OCD symptoms and model-based planning in an online sample, using a two-tailed test with a significance level of p<.05, the sample size should range between N=1223-1637.

**Exclusion criteria for online task data.** In line with suggestions made for conducting experiments online using Amazon’s Mechanical Turk (AMT), *a priori* exclusion criteria were applied to ensure data quality (Crump, McDonnell and Gureckis, 2013). Subjects were excluded if they missed more than 10% of trials (n=62), responded on the same key on more than 95% of trials on which they registered a response (n=85) or had implausibly fast reaction times, i.e. ±2 standard deviations from the mean (n=18). *Clinical Questionnaires Exclusion Criterion:* In an effort to identify participants who were not reading the questions prior to selecting their responses, we included one catch item: “If you are paying attention to these questions, please select "A little" as your answer”. Very few subjects failed to select the appropriate response to this catch question; those that did were excluded (n=6). *IQ Test Exclusion Criterion:* Participants who did not answer correctly to any of the IQ questions were excluded from further analysis (n=87). The adaptive character of the test meant that participants responding incorrectly received increasingly easy items; consistently failing to respond correctly indicates that given participants might have been inattentive or dishonest. In total, 258/1671 (15%) were excluded from this experiment, in line with a previously published report using this dataset. Note that in this dataset, it was also established that the results did not change regardless of the application of these criteria (Gillan *et al.*, 2016b). In addition to these criteria, we also required subjects to score 100% on a brief test that queried their comprehension of the task instructions. If they failed this test, they were required to restart the instructions (and repeat the practice trials) until the 100% criterion was reached.

**Panic Attacks and Life Stress**. The occurrence of recent panic attacks was assessed using item 1 on the self-report version of the Panic Disorder Severity Scale (PDSS): “How many panic and limited symptoms attacks did you have during the week?”. Subjects were provided with a definition of a panic attack: a “sudden rush of fear or discomfort”, peaking within 10 minutes accompanied by 4 of 17 symptoms (e.g. rapid or pounding heartbeat, feeling of choking, nausea, chills or hot flushes, fear of dying). Subjects were told that episodes that have fewer than 4 symptoms are ‘limited symptom attacks’. Panic attack frequency scores ranged from none (“no panic or limited symptom attacks”), mild (no full panic attacks and no more than 1 limited symptom attack/day), moderate (“1 or 2 full panic attacks and/or multiple limited symptom attacks/day”), severe (Severe: more than 2 full attacks but not more than 1/day on average) and extreme (“full panic attacks occurred more than once a day, more days than not”).

In the Social Readjustment Scale, events are weighted in a manner that reflects the relative amount of stress that event causes, with the death of a spouse and divorce being the most stressful and minor violations of the law, major holidays and vacations being the least.

**Control Variables.** As detailed in a prior report, subjects completed a range of self-report questionnaires that were the topic of a factor analysis in a previously published study (Gillan *et al.*, 2016a), which was subsequently validated in an independent dataset (Rouault *et al.*, 2018). One factor, titled “Compulsive Behaviour and Intrusive Thought”, was shown to be highly associated with model-based planning failures in this sample. Scores on this factor were thus controlled, along with IQ, age and gender.

**Detailed Results for Model-Based Task and Panic Attacks (past week).** Basic results from this task, and its association to compulsivity, age and IQ, have been published in detail elsewhere (Gillan *et al.*, 2016a). The novel results relevant to this study are as follows: one-trial-back regression analysis controlling for IQ, age and gender only, revealed that the frequency of panic attacks in the past week was associated with reductions in model-based planning (p=.012), and also increase in switch behavior (p=.04), but no effect on model-free learning (p=.80). Neither of these significant effects survived inclusion of compulsivity in the model (panic_attack*model-based, *p*=.33; panic_attack*switching, *p*=.24).

**Table S7. Results from Regression Analysis with Anxiety Attacks**

|  |  |  | | |
| --- | --- | --- | --- | --- |
| **Coefficient** | **β** | **SE** | ***z*-value** | ***p*-value** |
|  |  |  |  |  |
| **model-based * panic attack** | **-0.03** | **0.01** | **-2.52** | **.012*** |
| *controlling for compulsivity* | -0.01 | 0.01 | -0.97 | .33 |
| model-free * panic attack | -.005 | 0.02 | -0.25 | .80 |
| *controlling for compulsivity* | .004 | 0.02 | 0.223 | .82 |
| **repetition * panic attack** | **-0.07** | **0.04** | **-2.09** | **.04*** |
| *controlling for compulsivity* | -.04 | 0.04 | -1.19 | .23 |
|  |  |  | | |

**Detailed Results for Model-Based Task and Life Stress (12 months).**

**Table S8. Results from Regression Analysis with Life Stress (12 month)**

|  |  |  | | |
| --- | --- | --- | --- | --- |
| **Coefficient** | **β** | **SE** | ***z*-value** | ***p*-value** |
|  |  |  |  |  |
| **model-based * life stress** | **-.02** | **.01** | **-2.02** | **.04*** |
| *controlling for compulsivity* | -.01 | .01 | -.98 | .33 |
| model-free * life stress | -.01 | .02 | -.74 | .46 |
| *controlling for compulsivity* | -.01 | .02 | -.46 | .65 |
| repetition * life stress | -.02 | .03 | -.87 | .38 |
| *controlling for compulsivity* | -.01 | .03 | -.22 | .83 |
|  |  |  | | |

**Computational Modeling Method for Experiment 3**

The computational model proceeded exactly in Experiment 2, except that the within-subject manipulation was absent. We estimated each subject’s learning rate, model-based, model-free, perseveration and choice stochasticity parameters and then tested the extent to which these parameters were associated with panic attacks and life stress, after controlling for age, gender, IQ and the compulsive dimension in secondary regression analyses. The general pattern from the simpler analysis was reproduced with a couple of slight differences. First, the effect of panic attacks on model-based planning was not significant, even without controlling for compulsivity (Table S10). Second, the effect of panic attacks on choice switching (*p*) was significant both when compulsivity was and was-not controlled for (Table S10).

**Table S9. Association between having a recent panic attack (Item 1 on PDSS) and parameters in the computational model.**

|  |  |  | | |
| --- | --- | --- | --- | --- |
| **Coefficient** | **β** | **SE** | ***z*-value** | ***p*-value** |
| learning rate * panic attack | .00 | .01 | 0.25 | .81 |
| *controlling for compulsivity* | .01 | .01 | 0.88 | .38 |
| **perseveration * panic attack** | **-0.05** | **0.02** | **-2.55** | **.01**** |
| *controlling for compulsivity* | -0.04 | 0.02 | -1.87 | .06 |
| model-based * panic attack | -0.02 | 0.01 | -1.62 | .10 |
| *controlling for compulsivity* | -0.00 | 0.01 | 0.25 | .81 |
| model-free * panic attack | -0.01 | 0.03 | -0.32 | .75 |
| *controlling for compulsivity* | 0.01 | 0.03 | 0.30 | .76 |
| stochasticity * panic attack | -0.03 | 0.04 | -0.66 | .51 |
| *controlling for compulsivity* | -0.04 | 0.04 | 0.90 | .37 |

**Table S10. Association between Life Stress (12 months) on parameters in the computational model**

|  |  |  | | |
| --- | --- | --- | --- | --- |
| **Coefficient** | **β** | **SE** | ***z*-value** | ***p*-value** |
|  |  |  |  |  |
| learning rate * panic attack | -0.00 | .01 | -.02 | .98 |
| *controlling for compulsivity* | 0.00 | .01 | .36 | .72 |
| perseveration * panic attack | -0.00 | .02 | -.18 | .86 |
| *controlling for compulsivity* | 0.01 | .02 | .38 | .70 |
| **model-based * panic attack** | **-0.02** | **.01** | **-2.33** | **.02*** |
| *controlling for compulsivity* | -0.01 | .01 | -1.17 | .24 |
| model-free * panic attack | -0.02 | .02 | -0.61 | .54 |
| *controlling for compulsivity* | -0.01 | .03 | -0.22 | .82 |
| **stochasticity * panic attack** | **-0.08** | **.03** | **-2.43** | **.02*** |
| *controlling for compulsivity* | -0.05 | .03 | -1.52 | .13 |

**References**

Bellman, R. (1957) *Dynamic Programming.* Princeton, NJ: Princeton University Press.

Crump, M. J., McDonnell, J. V. and Gureckis, T. M. (2013) 'Evaluating Amazon's Mechanical Turk as a tool for experimental behavioral research', *PLoS One,* 8(3), pp. e57410.

Bürkner, PC(2017). brms: An R Package for Bayesian Multilevel Models Using Stan. *Journal of Statistical Software*, 80(1), 1-28.<[doi:10.18637/jss.v080.i01](https://doi.org/10.18637/jss.v080.i01)>.

Daw, N. D., Gershman, S. J., Seymour, B., Dayan, P. and Dolan, R. J. (2011) 'Model-Based Influences on Humans' Choices and Striatal Prediction Errors', *Neuron,* 69(6).

Dickinson, A. and Balleine, B. (1994) 'Motivational control of goal-directed action', *Animal Learning & Behavior,* 22(1), pp. 1-18.

Gelman, A. and Rubin, D. (1992) 'Inference from iterative simulation using multiple sequences', *Statistical Science,* 7(4), pp. 457-472.

Gillan, C., Kosinski, M., Whelan, R., Phelps, E. and Daw, N. (2016a) 'Characterizing a psychiatric symptom dimension related to deficits in goal-directed control', *eLife,* 5(e11305 ).

Gillan, C. M., Kosinski, M., Whelan, R., Phelps, E. A. and Daw, N. D. (2016b) 'Characterizing a psychiatric symptom dimension related to deficits in goal-directed control', *Elife,* 5.

Gillan, C. M., Otto, A. R., Phelps, E. A. and Daw, N. D. (2015) 'Model-based learning protects against forming habits', *Cognitive, Affective and Behavioral Neuroscience*.

Ito, M. and Doya, K. (2009) 'Validation of decision-making models and analysis of decision variables in the rat basal ganglia', *Journal of Neuroscience,* 29(31), pp. 9861-74.

Lau, B. and Glimcher, P. W. (2005) 'Dynamic response-by-response models of matching behavior in rhesus monkeys', *Journal of Experimental Analysis of Behavior,* 84(3), pp. 555-79.

Otto, A. R., Raio, C. M., Chiang, A., Phelps, E. A. and Daw, N. D. (2013) 'Working-memory capacity protects model-based learning from stress', *Proceedings of the National Academy of Sciences of the United States of America,* 110(52), pp. 20941-6.

Rouault, M., Seow, T., Gillan, C. M. and Fleming, S. M. (2018) 'Psychiatric Symptom Dimensions Are Associated With Dissociable Shifts in Metacognition but Not Task Performance', *Biological Psychiatry,* 84(6), pp. 443-451.

Rummery, G. and Niranjan, M. (1994) *On-Line Q-Learning Using Connectionist Systems.* Cambridge, UK: Cambridge University Press.

Schwabe, L. and Wolf, O. T. (2010) 'Socially evaluated cold pressor stress after instrumental learning favors habits over goal-directed action', *Psychoneuroendocrinology*.

Sharp, M. E., Foerde, K., Daw, N. D. and Shohamy, D. (2016) 'Dopamine selectively remediates 'model-based' reward learning: a computational approach', *Brain,* 139(Pt 2), pp. 355-64.

Sheehan, D. V., Lecrubier, Y., Sheehan, K. H., Amorim, P., Janavs, J., Weiller, E., Hergueta, T., Baker, R. and Dunbar, G. C. (1998) 'The Mini-International Neuropsychiatric Interview (M.I.N.I.): the development and validation of a structured diagnostic psychiatric interview for DSM-IV and ICD-10', *J Clin Psychiatry,* 59 (Suppl 20), pp. 22-33.

Stan (2014) *Stan: A C++ Library for Probability and Sampling, Version 2.5*. available from: <http://mc-stan.org/> (Accessed: October 15, 2014.

Voon, V., Derbyshire, K., Rück, C., Irvine, M. A., Worbe, Y., Enander, J., Schreiber, L. R., Gillan, C., Fineberg, N. A., Sahakian, B. J., Robbins, T. W., Harrison, N. A., Wood, J., Daw, N. D., Dayan, P., Grant, J. E. and Bullmore, E. T. (2014) 'Disorders of compulsivity: a common bias towards learning habits', *Molecular Psychiatry*.

Worbe, Y., Palminteri, S., Savulich, G., Daw, N. D., Fernandez-Egea, E., Robbins, T. W. and Voon, V. (2015) 'Valence-dependent influence of serotonin depletion on model-based choice strategy', *Moecular Psychiatry*.

Wunderlich, K., Smittenaar, P. and Dolan, R. J. (2012) 'Dopamine Enhances Model-Based over Model-Free Choice Behavior', *Neuron,* 75(3).
